# Data on genome analysis of *Mycoplasmagallisepticum* during intracellular infection

**DOI:** 10.1016/j.dib.2016.12.006

**Published:** 2016-12-08

**Authors:** Daria Matyushkina, Olga Pobeguts, Irina Garanina, Vladislav Babenko, Maria Vakhitova, Gleb Fisunov, Vadim Govorun

**Affiliations:** a Laboratory of Proteomic Analysis, Federal Research and Clinical Centre of Physical-Chemical Medicine, Moscow 119435, Russia; b Laboratory of Proteomics, Shemyakin-Ovchinnikov Institute of Bioorganic Chemistry, Moscow 117997, Russia; c Laboratory of Post-Genomic Research in Biology, Federal Research and Clinical Centre of Physical-Chemical Medicine, Moscow 119435, Russia; d Moscow Institute of Physics and Technology (State University), Dolgoprudny 141700, Russia

**Keywords:** Host-pathogen interaction, Mycoplasma, Genome, Vlh antiges, Nucleotide polymorphism

## Abstract

The genus *Mycoplasma* relates to Gram-positive bacteria that lack a cell wall and are capable to cause chronic disease in humans and animals. Among the agents of infection and disease in domestic poultry and wild birds, *Mycoplasma gallisepticum* is the most important mycoplasma species, causing considerable losses in the poultry industry. In the present paper, we provide data on adaptation of *M. gallisepticum* to the eukaryotic host cells on the genomic level. The major changes were predominantly localized in the VlhA-hemagglutinin genes which are important components of pathogenesis. The ability of mycoplasmas to change dramatically the repertoire of surface antigens and to vary the immunogenicity of these components allows them to remain undetected by the immune system of the host. The data presented in this article are related to the article entitled “Phase Transition of the Bacterium upon Invasion of a Host Cell as a Mechanism of Adaptation: a *Mycoplasma gallisepticum* Model.” (Matyushkina et al., 2016) [1]. Data posted in repository https://www.ncbi.nlm.nih.gov/bioproject/315515. Bioproject ID: PRJNA315515.

**Specifications Table**

**Table:** 

Subject area	*Biology*
More specific subject area	*Genomics*
Type of data	*Table*
How data was acquired	*Data was acquired on Ion Torrent PGM (Life Technologies) and 454 GS FLX+ (Roche)*
Data format	*Raw, processed*
Experimental factors	*Mycoplasma gallisepticum S6 cells were cultured as described previously*[2]*. Chicken erythroblast cell line HD3 (clone A6 of line LSCC*[3], [4]*) was cultivated as described in*[5]*. The gentamicin invasion assay and isolation of intracellular mycoplasma were carried out as described in*[1]*. Genomic DNA from individual clones was isolated as previously described*[2].
Experimental features	*Sequencing was performed according to Life Technologies and Roche protocols for DNA-seq.*
Data source location	*N/A*
Data accessibility	*Data is within this article and raw data was deposited at NCBI repository*https://www.ncbi.nlm.nih.gov/bioproject/315515*. Bioproject ID: PRJNA315515.*

**Value of the data**

• This data set will be of value for the scientific community working in the area of host-pathogen interaction since it represents the genome changes of bacterium *Mycoplasma gallisepticum* upon invasion of a host cell.
• The data will also be of value for studies in the area of infection and immunity because basic genome changes were predominantly localized in the VlhA-hemagglutinin genes which are the primary strategy for survival among bacterial pathogens.
• These data may have implications for the development of preventive strategies.

## 1 Data

The data represents the genomic polymorphisms of *Mycoplasma gallisepticum* clones after infection and isolation from HD3 cells. Table 1 represent data obtained during acute (24 h) infection. Table 2 represent data obtained during chronic (7 weeks) infection. In analysis were taken 10 different colonies of mycoplasma isolated from HD3 cells after acute infection, 10 different colonies of mycoplasma isolated from HD3 cells after chronic infection and 12 different colonies of control laboratory strain.

## 2 Experimental design, materials and methods

### 2.1 Cell culturing

*M. gallisepticum S6* cells were cultured as described previously [2]. Chicken erythroblast cell line HD3 (clone A6 of line LSCC [3], [4]) was cultivated as described in [5]. The gentamicin invasion assay and isolation of intracellular mycoplasma were carried out as described in [1]. Genomic DNA from individual cultures was isolated as previously described [2].

### 2.2 Genome sequencing and analysis

Genomic DNA from individual cultures was isolated as previously described [2]. The DNA (100 ng for each sample) was disrupted into 200–300 bp fragments using the Covaris S220 System (Covaris, Woburn, Massachusetts, USA). Barcode shotgun libraries for mycoplasma isolated from eukaryotic cells (MIEC) were prepared by the Ion Xpress™Plus Fragment Library Kit (Life Technologies). PCR emulsion was performed by the Ion PGM™Template OT2 200 Kit (Life Technologies). DNA sequencing was performed by the Ion Torrent PGM (Life Technologies) with the Ion 318 chip v2 and the Ion PGM™Sequencing 200 Kit v2 (Life Technologies). Control *M. gallisepticum S6* strain was sequenced by using the Roche 454 Life Sciences Genome Sequencer FLX following the manufacturer׳s instructions (Roche 454 Life Science, USA). Assembly of raw sequencing reads with an average length of 540 bases was performed by the GS de novo assembly software version 2.8 (Roche 454 Life Science, USA).

For the detection of nucleotide variants relatively to the reference, a reference-based mapping approaches via bowtie2 [6] and samtools mpileup [7] tools were used. On average 93% of reads mapped to the reference genome. We skipped alignments with mapping quality (mapQ) less than 10. Variants were called using the samtools mpileup command with options -C50 -D -S. Variants were filtered using the following criteria: (1) the depth of high-quality coverage larger than 20, (2) in average across all samples at least 50% of reads at the site supporting the call, (3) at least 5 samples have the variant, (4) a homozygous call under a diploid model. We identified nucleotide polymorphisms by comparing calls between the control genomes and the MIEC genomes.

## Figures and Tables

**Table 1:** Comparative genomic analysis of *M. gallisepticum* isolated from HD3 cells after acute (24 h) infection with laboratory strain of *M. gallisepticum S6.*

**ORF name**	**Gene name**	**Position**	**Ref**	**MIEC**	**Quality**	**Combined depth across samples**	**Mean allele frequency across samples**
GCW_00395	23S ribosomal RNA	83344	T	C	68.3	422	1
GCW_01160	VlhA.1.01 variable lipoprotein family protein	264070	C	T,A	999	239	1
GCW_01340	23S ribosomal RNA	317111	T	C	37.2	431	1
GCW_01345	5S ribosomal RNA	318386	G	A	999	479	1
GCW_01455	Upstream of mobile element protein	348358	A	T	999	229	0.8325
GCW_01960	VlhA, cluster 2	465075	A	G	999	92	1
		465216	T	A	55.7	128	1
GCW_92037	Phenylalanyl-tRNA synthetase (PheRS) beta chain core domain	483621	A	T,G	51.4	85	0.8354
GCW_92457	VlhA, cluster 3	588982	G	A,C	63.6	162	1
							
GCW_03335	VlhA, cluster 4	800656	T	C,A	999	482	1
		801100	T	C	999	666	1
						
GCW_93371		816612	G	T	999	121	1
		816905	A	T	999	595	1
		817621	G	T,A	999	762	1
		817733	A	G	999	935	1
		818171	C	A,G	20.9	99	1
GCW_93372	Upstream of VlhA, cluster 4	818493	T	G	999	449	1
GCW_00585	Serine protease	137223	G	A	999	836	0.6297
GCW_91948	Upstream of VlhA, cluster 2	462290	G	A	156	168	0.7728
GCW_92433	Upstream of VlhA, cluster 3	577485	G	T	999	384	0.6192
GCW_92454	VlhA, cluster 3	586155	G	T	999	238	0.5183
GCW_03035	30S ribosomal protein S12	722693	C	A	999	438	0.654
GCW_03140	Major facilitator superfamily permease	750922	G	C	999	489	0.6667
GCW_03470	Asparagine synthase	839170	A	G	999	907	0.6487

**MIEC** – mycoplasma isolated from eukaryotic cells; **Ref** – references strain of *M. gallisepticum S6.*

**Table 2:** Comparative genomic analysis of *M. gallisepticum* isolated from HD3 cells after chronic (7 weeks) infection with laboratory strain of *M. gallisepticum S6.*

**ORF name**	**Gene name**	**Position**	**Ref**	**MIEC**	**Quality**	**Combined depth across samples**	**Mean allele frequency across samples**
GCW_90633	Upstream of mobile element protein	152250	A	T,G	999	7417	1
GCW_01160	vlhA, cluster 1	264070	C	T,A	999	1465	1
GCW_01345	5S ribosomal RNA	318386	G	A	999	6909	1
GCW_01395	Asparaginyl-tRNA synthetase	332883	G	T	999	2712	0.9444
GCW_01520	M42 glutamyl-aminopeptidase family protein	359323	T	C	999	1107	0.9444
GCW_01960	vlhA, cluster 2	465075	A	G	999	2067	1
		465121	C	A,G	97.5	403	1
		465125	T	A	999	429	1
		465131	A	T,G	159	301	1
		465137	G	A	171	326	1
		465154	C	G	999	531	1
		465159	G	C	175	702	1
		465166	T	C	999	867	1
		465216	T	A	999	2494	1
GCW_92037	Phenylalanyl-tRNA synthetase (PheRS) beta chain core domain	483621	A	G,T	999	703	1
GCW_02455	Upstream of VlhA, cluster 3	588982	G	A	999	1135	1
GCW_03040	30S ribosomal protein S4	723019	T	C	999	6594	1
GCW_03045	Hypothetical protein DUF3682, eukaryotic protein	724257	G	C	999	7035	0.9444
GCW_03335	Upstream of VlhA family protein	801100	T	C	999	5441	1
GCW_93371	vlhA, cluster 4	816612	G	T	999	5428	1
		816905	A	T	999	6345	1
		817733	A	G	999	7904	1
		818171	C	A,G	999	1297	1
		818177	A	T,G	30.6	1407	1
		818179	C	T,G	50.5	1371	1
		818184	T	G	87.6	1185	1
		818186	T	A	77.6	1097	1
		818187	G	T	51.6	1038	1
		818188	G	T	88.5	1024	1
		818193	G	C	41.6	908	1
		818194	G	C	50.6	874	1
		818202	T	A	47.7	576	1
		818225	T	G	94.2	251	1
		818226	G	T,A	95.8	261	1
		818228	A	G	106	331	1
		818255	T	C,A	999	991	1

**MIEC** – mycoplasma isolated from eukaryotic cells; **Ref** – references strain of *M. gallisepticum S6.*
