# Supplementary material for: Data on genome analysis of Mycoplasmagallisepticum during intracellular infection
Source: Data Brief. 2016 Dec 8;10:264–8. doi: 10.1016/j.dib.2016.12.006 (PMC5217774; doi:10.1016/j.dib.2016.12.006)
Supplement: Supplementary file 1 — Supplementary material [file mmc1.docx]

Conflict of interest: The authors declare no conflict of interest.
